# Functional autonomic nervous system profile in children with autism spectrum disorder

**DOI:** 10.1186/2040-2392-5-39

**Published:** 2014-07-04

**Authors:** Azadeh Kushki, Jessica Brian, Annie Dupuis, Evdokia Anagnostou

**Affiliations:** 1 Bloorview Research Institute, 150 Kilgour Road, M4G 1R8 Toronto, Canada; 2 The Institute of Biomaterials and Biomedical Engineering, University of Toronto, 164 College Street, M5S 3G9 Toronto, Canada; 3 The Hospital for Sick Children, 555 University Avenue, M5G 1X8 Toronto, Canada

**Keywords:** Autism spectrum disorder, Autonomic nervous system, Heart rate, Respiratory sinus arrhythmia

## Abstract

**Background:**

Autonomic dysregulation has been recently reported as a feature of autism spectrum disorder (ASD). However, the nature of autonomic atypicalities in ASD remain largely unknown. The goal of this study was to characterize the cardiac autonomic profile of children with ASD across four domains affected in ASD (anxiety, attention, response inhibition, and social cognition), and suggested to be affected by autonomic dysregulation.

**Methods:**

We compared measures of autonomic cardiac regulation in typically developing children (n = 34) and those with ASD (n = 40) as the children performed tasks eliciting anxiety, attention, response inhibition, and social cognition. Heart rate was used to quantify overall autonomic arousal, and respiratory sinus arrhythmia (RSA) was used as an index of vagal influences. Associations between atypical autonomic findings and intellectual functioning (Weschler scale), ASD symptomatology (Social Communication Questionnaire score), and co-morbid anxiety (Revised Children’s Anxiety and Depression Scale) were also investigated.

**Results:**

The ASD group had marginally elevated basal heart rate, and showed decreased heart rate reactivity to social anxiety and increased RSA reactivity to the social cognition task. In this group, heart rate reactivity to the social anxiety task was positively correlated with IQ and task performance, and negatively correlated with generalized anxiety. RSA reactivity in the social cognition task was positively correlated with IQ.

**Conclusions:**

Our data suggest overall autonomic hyperarousal in ASD and selective atypical reactivity to social tasks.

## Background

Autism spectrum disorder(ASD) is a complex neurodevelopmental disorder defined by qualitative impairments in social communication, and by the presence of repetitive behaviors and restrictive interests. An extensive body of literature suggests that ASD symptoms are associated with pervasive atypicalities in the central nervous system [1], including structures and networks involved in the regulation of the autonomic nervous system (ANS). For example, ASD has been associated with atypicalities in the amygdala [2-5], the anterior cingulate cortex [6], and the insula [7,8] - structures that play a key role in modulation of the ANS response. It is therefore not surprising that, recently, a number of studies have reported atypicalities at the level of the peripheral nervous system, specifically pertaining to the dysregulation of the ANS [9]. These include reports of elevated basal heart rate [10-13], decreased baseline vagal tone [11-14], and blunted heart rate response to psychosocial challenges [15-17]. ANS indices have also been associated with core and co-morbid symptomatology in ASD. For example, decreased basal respiratory sinus arrhythmia (RSA) has been associated with difficulties in social behavior [14,18], decreased language abilities [18,19], internalizing symptoms [14], and cognitive delay [19].

Despite this early evidence of ANS dysregulation in ASD, wide variability in samples, methods, and measures has produced inconsistent literature findings [20]. As previously mentioned, the ANS has direct and indirect connections to a number of cortical and subcortical brain regions whose structure, function, and connectivity are affected in ASD (for example, prefrontal, limbic, and frontal networks, the hypothalamus, and the brainstem). Moreover, ANS function is closely linked to behavior, cognition, and emotion processing [20], and indices of its function have been associated with differences in domains affected in ASD [21] such as social behavior [22], emotion regulation [23], attention [24], response inhibition [25], and adaptive functioning [26]). Given the close association between the ANS and both the central nervous system and behavioral domains, ANS atypicalities, if found, can ultimately serve as non-invasive and inexpensive markers for deficits and co-morbidities associated with ASD. The objective of this study was to address the paucity of literature in understanding ANS dysregulation in ASD by characterizing the autonomic profile of children with ASD across four domains associated with ASD and affected by ANS dysregulation (anxiety, attention, response inhibition, and social cognition). We hypothesized that individuals with ASD would show hyperarousal of the ANS. This is supported by the high prevalence of co-morbid anxiety in ASD [27], the genetic, neurobiological, and pheonotypical overlap between the two disorders [28-31], and the findings of physiological hyperarousal in anxiety disorders [32-34]. We also expected atypical ANS reactivity to attention, response inhibition, and social cognition tasks in ASD based on previous reports associating ANS dysregulation with these behavioral domains [21,35-37].

## Methods

### Participants

**Figure 1:**
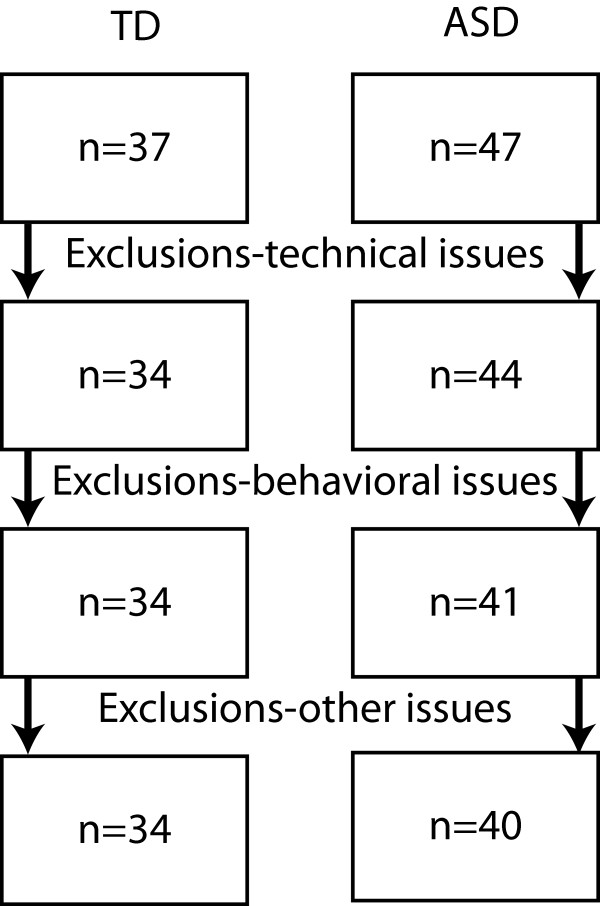
Participant numbers at different stages of the study.

We recruited a sample of typically developing (TD) children (n = 36) and children with ASD (n = 47) for the study (Figure 1). The inclusion criteria for the TD group were 8 to 18 years of age, no diagnosis of ASD or any other developmental, neurological, or psychiatric disorders, medication-free at the time of the study, and no premature birth (born after 35 weeks gestational age). The inclusion criteria for the ASD group were age 8 to 18 years, primary diagnosis of ASD, and ability to perform the study tasks. Children in the ASD group were diagnosed by an expert, research reliable team using the DSM-IV criteria supported by the Autism Diagnostic Observation Schedule (ADOS) [38] and the Autism Diagnostic Interview - Revised (ADI-R) [39].

The Bloorview Research Institute research ethics board approved the study. Participants deemed to have capacity for consent, provided written consent. For all other participants, assent and written consent were obtained from the children and their legal guardians, respectively.

### Procedures

After consent, participants completed a 2-hour study in one session. During the session, they sat in front of a computer screen and completed five tasks, each preceded and followed by a baseline task. The tasks were:

• Color Stroop (Color-Word Interference) test [40]. This task was used to elicit an ANS stress reaction [41,42], and shown to elicit both psychological and physiological arousal responses in typically developing populations [43] as well as children with ASD [10]. Participants completed a computerized, single-trial version of the task which involved the presentation of words corresponding to color names, printed in differently colored letters. The participants were required to name the color of the letters while ignoring the printed word. The task consisted of 5, one-minute blocks in which stimulus presentation frequency varied from 2 to 1.25 seconds/word (blocks one and five: 2 seconds/word, blocks 2 and 4: 1.5 seconds/word, block 5: 1.25 seconds/word). During the first and last blocks, only congruent stimuli were presented, whereas the remaining blocks consisted of only incongruent stimuli. Prior to starting the task, participants were provided with ten practice stimuli to ensure understanding of the task. Performance on this task was measured as the percentage of correct responses.

• Public speaking. For this task, participants were given 2 minutes to prepare a 3-minute talk on a topic of their choice. The talk was then delivered to three strangers. Public speaking tasks have been successfully used in previous studies examining cardiac responses to anxiogenic stimuli in neurotypical individuals [44-46] and in children with ASD [15,47]. Performance on this task was measured as the percentage of the time that the participant did not speak or vocalize during the task.

• Rapid visual information processing (CANTAB, http://www.camcog.com/). In this test of sustained attention and working memory [48], participants were presented with random single-digit numbers (2 through 9) at a rate of 100 digits per minute and asked to identify a prespecified three-digit sequence (for example, 3-5-7) by pressing the space bar. The duration of this task was 4 minutes. Performance was measured as the percentage of correctly identified sequences.

• Stop signal task [49]. This was a test of response inhibition. Participants were presented with a series of X’s and O’s and asked to press the left and right buttons on a gamepad when X’s and O’s appeared, respectively. The stimuli were occasionally followed by an auditory tone (stop signal), requiring the participants to withhold their response. The task consisted of 5 blocks, with 24 trials per block. The first block was practice. Total length of task was approximately 10 minutes. Task performance was measured as the latency of the stop process (stop signal reaction time (SSRT)) in milliseconds [50].

• Reading the Mind in the Eyes - child version [51]. This was a test of social cognition (theory of mind) where participants were presented with a set of 28 photographs depicting the eye region of the face, and asked to choose the word that best described what the person was thinking or feeling from a set of four choices. Performance was measured as the number of correct responses.

For the baseline task, participants watched clips of animated movies. All baseline tasks were 5 minutes in duration, except for the initial baseline which was 15 minutes long to allow acclimation to the environment and sensors.

### Measures

ASD symptom severity was further characterized using the Social Communication Questionnaire (SCQ). The parent version of the Revised Child Anxiety and Depression Scale (RCADS-P) [52] was used to characterize anxiety symptomatology in this group along scales corresponding to five DSM-IV anxiety disorders (separation anxiety disorder, social phobia, generalized anxiety disorder, obsessive-compulsive disorder, and panic disorder). Given the nature of our tasks, only scales corresponding to generalized anxiety disorder and social phobia were considered in the analyses. Intellectual functioning was assessed in both groups using the Wechsler Scales of Intelligence (Wechsler Abbreviated Scale of Intelligence (I and II), and the Wechsler Intelligence Scale for Children IV). For one participant in the TD group, an existing intelligence score (the Stanford-Binet Intelligence Scale) was used.

A three-lead electrocardiogram (ECG) was measured using a wearable sensor from Shimmer Research. The ECG time series, sampled at 256 Hz, was transmitted over BlueTooth to a laptop computer and stored. Analyses were carried out offline using Matlab. Heart rate (HR) was calculated as the inverse of inter-beat (RR) intervals extracted using a modified version of the algorithms presented in the works [53,54]. In particular, the ECG signal was bandpass filtered between 5 and 15 Hz to maximize the QRS energy and to remove artifact noise, including baseline wandering, motion artifacts, and electrical noise. The signal was then differentiated, squared, and integrated using a 200-ms window. Peaks of the integrated signals were detected as R-waves using a detection threshold of 0.15 times the median of the past ten beats and a blanking period of 200 ms. The RR sequence then underwent an outlier filtering algorithm which removed RR values outside of acceptable limits. High and low limits were computed as 75% and 150% of the average of the median of all beats and the median of eight preceding beats. This combination was used to allow for adaptation to changes in median RR-interval over time. The ensuing analyses used RR values from the last 3 minutes of each task interval.

To compute RSA, each RR sequence was uniformly sampled at 8 Hz using cubic interpolation and filtered using a third-order, 21-point polynomial filter to remove the variance associated with activities at frequencies lower than spontaneous breathing [55,56]. The filtered signal was then subtracted from the original resampled sequence and the power spectral density of this residual sequence was estimated using Welch’s averaged modified periodogram method (Hamming window, 800-point Fourier transform).

RSA was calculated as the natural logarithm of the total power (that is, the summation of the spectral components) in the frequency band 0.24 to 1.04 Hz, which is used in studies of vagal functioning in children [57]. The analyses were repeated using the frequency band 0.15 to 0.40 Hz [58], but the conclusions remained unchanged.

### Statistical analyses

Statistical analyses were performed using SAS version 9.4 (SAS Institute, Cary, NC). To investigate group differences in HR and RSA, repeated measures multiple regression analysis was used to examine the significance of group × task interaction using individual models for each measure. Sex, IQ, and age were included as covariates in these models. For RSA, mean baseline HR was also included as a covariate [59]. Using these models, we examined group differences in baseline HR and RSA, as well as reactivity in these measures in response to each task using contrast statements. In post hoc analyses, stepwise regression was used to examine whether baseline and reactivity cardiac measures were significantly predicted by demographic variables (age, full-scale IQ, sex), autism symptomatology (SCQ), trait anxiety (RCADS scores on the generalized anxiety disorder and social phobia subscales), and task performance. Stepwise regression combined forward selection and backward elimination with alpha-to-enter and alpha-to-remove set to 0.1.

We tested for group differences on task performance using generalized linear models, which can be used for non-normally distributed errors, adjusting for age, sex, and full-scale IQ. Outcomes were modeled for the normal, Poisson, zero-inflated Poisson, and zero-inflated negative binomial distribution as appropriate.

For all analyses, if a participant did not complete a task, the data from that task were treated as missing but the data from remaining tasks were used in the analysis.

## Results

### Participants

Due to technical difficulties with the data collection software, two participants in the TD group and three participants in the ASD group were excluded from the analyses. Three participants with ASD refused to comply with study protocol and were excluded. Data from an additional participant in the ASD group were excluded because of a fire alarm during the session. The demographic information for the remaining participants is shown in Table 1, and participant numbers during different stages of the study are shown in Figure 1.

**Table 1:** Participant characteristics

	**TD (n = 34)**	**ASD (n = 40)**	**P value (group effect)**
Age	12.5 ± 2.9	12.0 ± 2.9	0.5
Full-scale IQ	113.1 ± 13.9	92.9 ± 20.6	< 0.0001
Sex (male:female) ^∗^	19:15	33:7	0.02
SCQ		18.0 ± 8.1	
RCADS-SAD		61.9 ± 17.4	
RCADS-GAD		58.6 ± 16.3	
RCADS-PD		57.3 ± 17.5	
RCADS-SP		53.1 ± 11.3	
RCADS-OCD		54.1 ± 10.5	
RCADS-total anxiety		58.4 ± 14.8	

^*^Fisher’s exact test.

RCADS: Revised Child Anxiety and Depression Scale; SCQ: Social Communication Questionnaire; SAD: separation anxiety disorder; GAD: generalized anxiety disorder; PD: panic disorder; SP: social phobia; OCD: obsessive-compulsive disorder.

Of the 40 children in the ASD group, 8 were taking medications at the time of the study. These included serotonin norepinephrine reuptake inhibitors (SNRIs) (Strattera), selective serotonin reuptake inhibitors (SSRIs) (Prozac, Zoloft, citalopram), stimulants (Ritalin, Biphentin, Concerta), and atypical antipsychotics (risperidone, Abilify). Co-morbid conditions reported by participants in the ASD group included attention deficit hyperactivity disorder (ADHD) symptoms (6), anxiety symptoms (4), developmental coordination disorder (2), history of hydrocephalus (1), dyslexia (1), and learning disability (1). One participant in the ASD group was born at 34 weeks.

The RR outlier filtering algorithm retained an average of 98.6% ± 2.2% of participant data. More than 5% of the data were removed for one participant in the TD group (10% removed) and two participants in the ASD group (9% and 13%). Exclusion of these participants did not change any of the conclusions reported in the following sections.

### Task performance

Table 2 reports the performance results for both the TD and ASD groups. After controlling for age, sex, and full-scale IQ, the performance of children in the ASD group was not significantly different than that of the TD group on any of the five tasks in this study (Table 3).

**Table 2:** Task performance for the typically developing (TD) and autism spectrum disorder (ASD) groups (mean ± standard deviation)

	**TD**	**ASD**
Stroop task (% correct)	85.5 ± 17.9	67.6 ± 30.8
Public speaking (seconds silence)	37.6 ± 40.6	65.2 ± 51.3
RVP (% correct)	91.8 ± 11.2	81.2 ± 19.4
Stop task (SSRT (ms))	325.0 ± 110	214.5 ± 86
Reading the Mind in the Eyes (# correct)	23.4 ± 3.1	20.1 ± 4.6

**Table 3:** **Comparison of performance between groups after controlling for IQ, age, sex (mean *± * standard error)**

	**TD**	**ASD**	**P value (group effect)**
Stroop task (% correct)	85.3 ±2.1	80.8 ± 2.6	0.18
Public speaking (log(seconds silence))	3.2 ± 0.2	3.6 ± 0.2	0.21
RVP (% correct)	85.1 ± 2.4	84.5 ± 2.5	0.87
Stop task (SSRT (ms))	339.0 ± 18.1	306.8 ± 19.0	0.25
Reading the Mind in the Eyes (# correct)	18.8 ± 0.5	18.6 ±0.5	0.73

### HR

**Figure 2:**
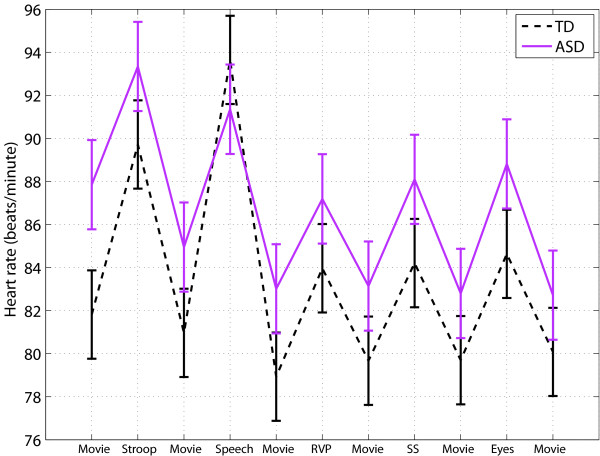
**HR: Mean across tasks for the TD and ASD groups.** Error bars represent standard error.

The mean HR for each task is shown in Figure 2 for the TD and ASD groups. For HR, repeated measures analysis revealed a significant group × task interaction (F(10,711) = 4.79, P < 0.0001). The ASD group had significantly higher baseline (movie 1) HR (estimated group difference = 6.02 ± 3.03 beats/minute, t(711) =-1.99, P = 0.047). The ASD group also had significantly decreased HR reactivity during the public speaking task (estimated group difference (speaking - pre baseline) =6.28 ± 1.31 beats/minute, t(711) = 4.79, P < 0.0001; estimated group difference (speaking - post baseline) =6.38 ± 1.31 beats/minute, t(711) = 4.87, P < 0.0001). Exclusion of the ASD participants on medications increased the P value for the baseline group difference (estimated group difference = -5.88 ± 3.19 beats/minute, t(632) = -1.85, P = 0.066). No significant group differences were found for HR reactivity in the Stroop, rapid visual information processing, stop signal, or Reading the Mind in the Eyes tasks.

### RSA

**Figure 3:**
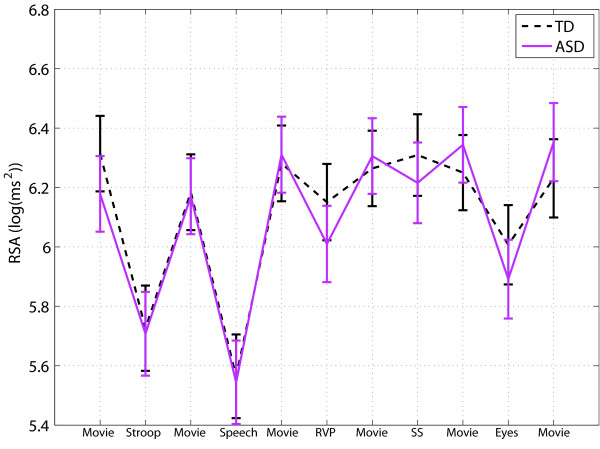
**RSA: Mean across tasks for the TD and ASD groups.** Error bars represent standard error.

The mean RSA for each task is shown in Figure 3 for the TD and ASD groups. Repeated measures analysis did not show a significant group × task interaction on RSA, and there was no significant difference in baseline RSA between the groups. However, in response to the Reading the Mind in the Eyes task, there was a marginally larger decrease in RSA in the ASD group (estimated group difference (Eyes - pre baseline) = 0.21 ± 0.11 log(ms ^2^), t(708) = 1.8, P = 0.07) and a larger RSA increase during recovery from the Reading the Mind in the Eyes task (estimated group difference (Eyes - post baseline) =0.24 ± 0.12 beats/minute, t(708) = 1.9, P = 0.06). Exclusion of the medication group decreased the P value (estimated group difference (Eyes - pre- baseline) =0.22±0.12 log(ms ^2^), t(629) = 2.15, P = 0.03; estimated group difference (Eyes - post baseline) = 0.25 ± 0.13 beats/minute, t(629) = 1.88, P = 0.06). This also revealed a marginally significant group difference in recovery from the stop signal task (estimated group difference (stop task - post baseline) = 0.25 ± 0.13 log(ms ^2^), t(629) = 1.94, P = 0.05), and in change and recovery from the RVP task (estimated group difference = 0.2 ± 0.11 log(ms ^2^), t(629) = 1.74, P = 0.08). No significant group differences were found for RSA reactivity in the Stroop, public speaking, or rapid visual information processing tasks.

### Association with demographic, anxiety, and performance measures

Table 4 summarizes the results of the post hoc correlation analysis examining associations between HR and RSA, and demographic, anxiety, and performance measures in the ASD group. The analyses were conducted for measures/task pairs for which significant group differences were revealed by the repeated measures analyses in the previous sections. After removing the medication group, stepwise regression revealed that the only significant predictor of baseline HR was age (model R-square = 0.14). HR change (speaking - pre baseline) was predicted by full-scale IQ and RCADS generalized anxiety score (model R-square = 0.36; partial R-squares: full-scale IQ = 0.20, generalized anxiety = 0.17). HR recovery during public speaking (speaking - post baseline) was predicted by full-scale IQ, RCADS generalized anxiety score, and performance on the task (model R-square = 0.46; partial R-squares: full-scale IQ = 0.15, generalized anxiety = 0.18, performance = 0.12).

**Table 4:** Associations between cardiac variables and demographic, anxiety, and performance measures for the ASD group

	**Age**	**IQ**	**RCADS GAD**	**Performance**
Baseline HR	-1.57 ± 0.70 ^∗^			
HR speaking-pre baseline		0.18 ± 0.05 ^∗∗^	-0.15 ± 0.06 ^∗^	
HR speaking-post baseline		0.19 ± 0.06 ^∗∗^	-0.18 ± 0.06 ^∗^	0.08 ± 0.04 ^∗^
RSA Eyes-pre baseline		-0.02 ± 0.005 ^∗∗^		
RSA Eyes-post baseline		-0.01 ± 0.005 ^∗^		

Only significant regression slopes (***β***) are reported. ^∗^P ***<*** 0.05, ^∗∗^P ***<*** 0.005. HR: heart rate; RSA: respiratory sinus arrhythmia; speaking: public speaking task; Eyes: Reading the Mind in the Eyes task; RCADS: Revised Child Anxiety and Depression Scale; GAD: generalized anxiety disorder.

The only significant predictor of RSA change during the Reading the Mind in the Eyes task was full-scale IQ (Eyes - pre baseline model R-square = 0.29; Eyes - post baseline model R-square = 0.20).

## Discussion

Our results suggest atypical cardiac findings in the ASD group despite group differences on task performance. These findings include cardiac hyperarousal in the ASD group as well as atypical responses to the public speaking and Reading the Mind in the Eyes tasks. In particular, while not statistically significant, we found that the ASD group had an elevated heart rate during the experimental session. Moreover, this group showed a blunted heart rate response to public speaking which was correlated with full-scale IQ and generalized anxiety score, and an exaggerated RSA response to the Reading the Mind in the Eyes task which was correlated with full-scale IQ.

Our data also showed that medication effects accounted for a marginal difference in the outcomes. This suggests that future studies on ANS functioning should report on the use of medications in their samples and account for their potential effects on ANS measures.

### Task performance

We did not find any group differences in task performance after adjusting for age, sex, and full-scale IQ. In the absence of such differences, ANS atypicalities may suggest compensatory mechanisms applied by the ASD group.

Reflecting the heterogeneity in the autism spectrum, there is considerable variability in the literature regarding performance on the tasks used in this study. For example, regarding the Stroop and stop signal tasks, several studies have reported no performance differences whereas others have reported significant group effects (see [60] for a review). With regard to public speaking, no group differences were found in the length of the public speech between ASD and TD participants (although physiological differences were found) [15]. With regard to the Reading the Mind in the Eyes Task, our results are adjusted for IQ, age, and sex. Our unadjusted means for the TD and ASD groups are well in line with the literature which has reported significant group differences [61]. Our analysis of task performance differences may be limited by the fact that the ASD and TD were not matched on IQ, as this may have reduced the statistical power to detect significant group differences. Future studies with larger sample sizes are needed to further investigate these differences.

### Baseline differences

Previous literature on cardiac measures of ANS have reported either hyperarousal [10-13,62] or no baseline atypicalities in ASD [47]. Consistent with the hyperarousal hypothesis, our data indicate that children with ASD had marginally significantly elevated HR at baseline compared to the typically developing children. These results replicate our own findings of basal hyperarousal in [10]. This hyperarousal trend persisted through the four domains examined in this study, although the difference did not reach statistical significance. This hyperarousal may be associated with co-morbid anxiety in ASD, a feature of ASD itself, or a manifestation of the interaction of ASD and anxiety symptoms.

• **Co-morbid anxiety in ASD:** Anxiety disorders are one of the most prevalent co-morbidities in ASD [27] and may exacerbate or occasionally drive the core deficits of ASD [30]. While the nature of anxiety in ASD is still largely unknown [63], ASD has been associated with genetic [28,29], neurobiological, and phenotypical overlap with anxiety disorders [30,31]. From a neurobiological perspective, both ASD and anxiety disorders have been associated with differences in central structures involved in emotional processing (such as the amygdala, anterior cingulate cortex, prefrontal cortex, and the insular cortex) [64,65]. In terms of phenotypic presentation, ASD and anxiety are thought to present with overlapping symptoms related to repetitive and restrictive interests (for example, perseverative behavior), social and emotional reciprocity, avoidance behaviors, and speech difficulties [31,63,66]. Interestingly, subclinical ASD traits have been reported in children with anxiety disorders [31].

  The clinical presentation of anxiety is conceptualized as having three interconnected dimensions: behavioral (such as crying, avoidance, or tantrums), subjective-cognitive (such as maladaptive or negative thoughts), and physiological (such as increased heart rate or perspiration) [67,68]. The latter often manifests itself as a "fight or flight" response which is associated with excitation and inhibition of the sympathetic and parasympathetic branches of the ANS, respectively. Highlighting the role of the physiological dimension, chronically elevated HR has been reported in anxiety disorders [32-34]. A similar pattern was found in the present study, suggesting physiological symptom overlap between ASD and anxiety disorders. As such, the hyperarousal found herein may reflect the cardiac signs of co-morbid anxiety.

  In this study, we did not find a significant correlation between baseline HR and trait anxiety measured by the RCADS. Previous literature findings on this association are mixed [45].

• **Hyperarousal as a feature of ASD:** ANS hyperarousal may also be related to neurobiological differences associated with ASD. Although the ANS response is modulated both at the central and peripheral levels, in the absence of any evidence indicating primary ANS dysfunction in ASD, we suggest that hyperarousal of the ANS system may be secondary to hyperarousal at the central level. At this level, hyperarousal may be associated with over-responsivity of networks involved in perception, processing, and responding to emotional stimuli [34]. Processes that may contribute to this over-responsivity include increased threat perception, increased and misinterpreted perception of bodily sensations [34], or decreased inhibition of the fear response. ASD has been associated with neurobiological differences in brain networks responsible for these functions (for example, the prefrontal cortex, the insular cortex, and the anterior cingulate cortices) [6], and aspects of these differences have been associated with anxiety symptom severity [69,70]. For example, total and right amygdala volumes have been positively associated with scores on the anxiety subscale of the Child Behavior Checklist [69] in a sample of children with ASD. At the same time, ASD has been associated with enlargement of the amygdala [2-4]. While these findings further support the role of the central nervous system in ANS hyperarousal, neuroimaging studies are needed to examine this hypothesis.

• **Interaction of ASD and anxiety features:** The observed hyperarousal in our sample may also be related to an interaction between anxiety and ASD features. For example, ASD features such as sensory sensitivities, insistence on sameness, or other affective and cognitive deficits may lead to increased stressors experienced by this population, which can ultimately lead to increased arousal [63,71]. Moreover, neurological dyregulation (for example, in threat perception or in introception) may further increase the susceptibility to conditioning by negative experiences and hyperarousal.

We did not find evidence of reduced parasympathetic tone as measured by RSA. Literature findings on this issue are divergent, with a number of studies reporting significantly reduced RSA [11-14], and others finding no atypicalities [47,62]. This discrepancy may be related to methodological differences, age-related changes (for example, developmental trend), and other confounding variables (anxiety, attention, level of intervention received). For example, our study was different than [11,12,14] in the baseline task used (such as movie watching versus sitting quietly), suggesting that task conditions may play a role in measurement of baseline ANS arousal. Future well-controlled studies are needed to further investigate these issues.

### Differences in task reactivity

Our data showed a blunted HR response to the social anxiety task in the ASD group. This pattern has been reported consistently in three studies examining ANS reactivity to psychosocial challenges [15-17], and was previously suggested to be a manifestation of response saturation due to basal hyperarousal. Consistent with this hypothesis, we found a significant negative association between HR change and scores on the generalized anxiety subscale of the RCADS. This atypical response, however, has not been previously reported in ANS studies of public speaking involving social phobics, and may therefore be unique to ASD. Interestingly, the blunted response was not seen in our second anxiety task (Stroop Color-Interference task). Given that impairments in social interaction are a defining feature of ASD, we hypothesize that the atypical response found in this study may reflect differences in subjective experiences of the task (such as motivation, attention, or judgement of the social threat). This hypothesis is consistent with our finding of a positive correlation between HR change, and IQ and task performance [63,72].

Our data also revealed increased RSA reactivity to the Reading the Mind in the Eyes task in the ASD group. This task is a test of theory of mind (ToM) or the ability to recognize others’ emotions and states of mind. A domain affected in ASD, ToM allows for fostering of appropriate social behaviors, establishment of reciprocal relationships, and development of social competence [12]. Social function, and ToM in particular, have also been linked to RSA regulation, as the "vagal brake" promotes calm behavioral states that are needed for social function, with greater ability to reduce RSA associated with better outcomes [21]. While we did not find an association between severity of social communication symptomatology as measured by the SCQ, our data did show that IQ was a significant predictor of RSA change in the ASD group. In the absence of performance differences between the groups, large RSA variability may reflect higher degree of effort or compensatory mechanisms applied by the ASD group. The latter is consistent with atypical central nervous system responses to the Eyes task (for example, decreased amygdala activation) [73].

### Limitations

Our data suggest basal hyperarousal in the ASD group. However, our baseline condition (movie watching) may not reflect a true "rest" state, and the pattern of hyperarousal at baseline may reflect increased ANS lability to laboratory conditions. Considering that we did not find HR over-reactivity to our experimental tasks, this scenario is unlikely.

Our sample size may have limited our ability to detect significant group differences in reactivity to attention and response inhibition tasks. Moreover, this sample hindered the analysis of subgroups that may exhibit different physiological responses (hyperarousal versus hypoarousal). Additionally, while our typically developing group was screened based on medical history, we were unable to report on the presence of any undiagnosed ASD traits in this population (for example, by using the SCQ).

Finally, the ANS has complex and bidirectional interactions with the central nervous system and the endocrine system. As such, our measures of cardiac autonomic control reflect the final common pathway of these interactions.

## Conclusions

Recently, it has been suggested that ASD may be associated with autonomic dysregulation. Our data are consistent with this hypothesis. In particular, we found basal heart rate hyperarousal and aytpical heart rate and respiratory sinus arrhythmia reactivity to two social tasks (public speaking and Reading the Mind in the Eyes). Future studies are needed to examine the central nervous system correlates of this dysregulation, as well as its association with co-morbid anxiety and ASD symptomatology. The finding of ANS dysregulation may ultimately suggest new treatment targets in ASD, and lead to inexpensive and non-invasive markers for deficits associated with this disorder.

## Abbreviations

ASD: Autism spectrum disorder; ANS: Autonomic nervous system; ECG: Electrocardiogram; HR: Heart rate; RCADS: Revised Child Anxiety and Depression Scale; RSA: Respiratory sinus arrhythmia; SCQ: Social Communication Questionnaire; SSRT: Stop signal reaction time; TD: Typically developing; ToM: Theory of mind.

## Competing interests

AK, JB, and AD declare that they have no competing interests. EA has received consultation fees from Novartis and Seaside Therapeutics and an unrestricted grant by Sanofi Canada.

## Authors’ contributions

AK conceptualized and designed the study, carried out the analyses, and drafted the manuscript. JB contributed to conceptualization and design of the study and interpretation of the results, supervised all psychological assessments, and edited the manuscript. AD contributed to the statistical analysis and edited the manuscript. EA contributed to conceptualization and design of the study, interpretation of the results, and drafting of the manuscript. All authors read and approved the manuscript.
